# The Effects of Expressing Empathy/Autonomy Support Using a COVID-19 Vaccination Chatbot: Experimental Study in a Sample of Belgian Adults

**DOI:** 10.2196/41148

**Published:** 2023-05-08

**Authors:** Wojciech Trzebiński, Toni Claessens, Jeska Buhmann, Aurélie De Waele, Greet Hendrickx, Pierre Van Damme, Walter Daelemans, Karolien Poels

**Affiliations:** 1 Collegium of Management and Finance SGH Warsaw School of Economics Warszawa Poland; 2 Department of Communication Studies University of Antwerp Antwerp Belgium; 3 Department of Linguistics University of Antwerp Antwerp Belgium; 4 Vaccine & Infectious Disease Institute University of Antwerp Antwerp Belgium

**Keywords:** COVID-19, vaccinations, chatbot, empathy, autonomy support, perceived user autonomy, chatbot patronage intention, vaccination intention, conversational agent, public health, digital health intervention, health promotion

## Abstract

**Background:**

Chatbots are increasingly used to support COVID-19 vaccination programs. Their persuasiveness may depend on the conversation-related context.

**Objective:**

This study aims to investigate the moderating role of the conversation quality and chatbot expertise cues in the effects of expressing empathy/autonomy support using COVID-19 vaccination chatbots.

**Methods:**

This experiment with 196 Dutch-speaking adults living in Belgium, who engaged in a conversation with a chatbot providing vaccination information, used a 2 (empathy/autonomy support expression: present vs absent) × 2 (chatbot expertise cues: expert endorser vs layperson endorser) between-subject design. Chatbot conversation quality was assessed through actual conversation logs. Perceived user autonomy (PUA), chatbot patronage intention (CPI), and vaccination intention shift (VIS) were measured after the conversation, coded from 1 to 5 (PUA, CPI) and from –5 to 5 (VIS).

**Results:**

There was a negative interaction effect of chatbot empathy/autonomy support expression and conversation fallback (CF; the percentage of chatbot answers “I do not understand” in a conversation) on PUA (PROCESS macro, model 1, B=–3.358, SE 1.235, *t*_186_=2.718, *P*=.007). Specifically, empathy/autonomy support expression had a more negative effect on PUA when the CF was higher (conditional effect of empathy/autonomy support expression at the CF level of +1SD: B=–.405, SE 0.158, *t*_186_=2.564, *P*=.011; conditional effects nonsignificant for the mean level: B=–0.103, SE 0.113, *t*_186_=0.914, *P*=.36; conditional effects nonsignificant for the –1SD level: B=0.031, SE=0.123, *t*_186_=0.252, *P*=.80). Moreover, an indirect effect of empathy/autonomy support expression on CPI via PUA was more negative when CF was higher (PROCESS macro, model 7, 5000 bootstrap samples, moderated mediation index=–3.676, BootSE 1.614, 95% CI –6.697 to –0.102; conditional indirect effect at the CF level of +1SD: B=–0.443, BootSE 0.202, 95% CI –0.809 to –0.005; conditional indirect effects nonsignificant for the mean level: B=–0.113, BootSE 0.124, 95% CI –0.346 to 0.137; conditional indirect effects nonsignificant for the –1SD level: B=0.034, BootSE 0.132, 95% CI –0.224 to 0.305). Indirect effects of empathy/autonomy support expression on VIS via PUA were marginally more negative when CF was higher. No effects of chatbot expertise cues were found.

**Conclusions:**

The findings suggest that expressing empathy/autonomy support using a chatbot may harm its evaluation and persuasiveness when the chatbot fails to answer its users’ questions. The paper adds to the literature on vaccination chatbots by exploring the conditional effects of chatbot empathy/autonomy support expression. The results will guide policy makers and chatbot developers dealing with vaccination promotion in designing the way chatbots express their empathy and support for user autonomy.

## Introduction

### Background

Chatbots are used as a promising tool to promote COVID-19 vaccinations [1,2] as they offer the possibility of upscaled interactions with users. Chatbots can also support health communication by engaging users via social media channels [3]. Unfortunately, there is a considerable amount of resistance toward COVID-19 vaccinations in society [4,5], and chatbots themselves may evoke negative user responses (eg, [6]). Specifically, the effects of chatbot empathy have been found to be ambiguous. On the one hand, empathy displayed by a chatbot (ie, a chatbot showing its understanding of and care for the user’s experience) may lead to a positive user response [7]. It may also enhance autonomy support (ie, provide the user with a sense of freedom in acting upon the information received from the chatbot), which is considered to diminish psychological reactance [8,9]. On the other hand, previous studies [6,10,11] suggest that the consequences of artificial agents’ empathy may also be negative. These studies focus on the way of expressing empathy, suggesting that being too “humanlike” is harmful. However, the role of the context related to the conversation, as conversation quality and chatbot expertise cues, remains understudied. Since that conversation-related context is manageable, this gap is also important from the practical perspective of vaccination chatbots. Therefore, this study aims to bridge this gap by investigating how conversation quality and chatbot expertise cues moderate the effect of expressing empathy/autonomy support using a COVID-19 vaccination chatbot. We propose that the effect of expressing empathy/autonomy support is less positive (or more negative) when the actual answers provided by the chatbots fail to dissolve queries submitted by users, because users may have higher expectations for the quality of interaction if a chatbot is interacting in a more human way [12,13]. Given the technical complexity of chatbot development, especially in the context of topics that require a lot of domain-specific and rapidly changing knowledge, such as the COVID-19 vaccines, conversation fallback (CF, ie, the situation in which a chatbot is unable to answer a user’s question) is plausible [14,15]. In addition, users may react more positively to the chatbot empathy/autonomy support expression when they perceive the chatbot to be more trustworthy and competent [16], which is relevant to a controversial topic, such as vaccinations. Therefore, we considered an expertise cue in the form of the chatbot endorser's credibility as an additional moderator of the empathy/autonomy support expression effect. We examined these moderating effects in an experimental study with different levels of expression of empathy/autonomy support and endorser credibility for a COVID-19 vaccination chatbot (VaccinChat), considering the actual level of conversation quality.

According to the Behavioral Intervention Technology Model [17], our chatbot (VaccinChat) can be considered a behavioral intervention technology whose clinical aim is to increase users’ intention to get vaccinated against COVID-19. The chatbot patronage intention (CPI, including the willingness to use and recommend the chatbot) is considered the usage aim. The behavioral intervention strategy is education (ie, providing information about how vaccines work and how a user can get vaccinated). Accordingly, the chatbot endorser credibility (expert vs layperson) can be considered a relevant expertise cue contributing to the user's initial trust toward the chatbot as an educational tool. Behavioral intervention elements are the pieces of vaccine-related information provided by the chatbot. They can be characterized as having a textual form, being instantly delivered online, and being personalized based on user queries formulated in natural language. The expression of empathy/autonomy support is an additional characteristic that is experimentally manipulated and varies based on specific questions asked by a user. The intervention workflow is user defined (ie, a user may decide whether and how frequently to reuse the chatbot). However, our experimental setting included a single intervention (ie, the chatbot was used only once by each study participant).

### Theoretical Development

#### Supporting Vaccinations and the Role of Chatbots

Even amid the COVID-19 pandemic threat, vaccine hesitancy, fueled by misinformation and conspiracy theories, remains an issue in health promotion efforts [5]. At the end of 2021 (about 1 year after making COVID-19 vaccines available), less than 69% of European Union (EU) citizens were vaccinated against COVID-19 (primary course), with even much lower values in some countries such as Bulgaria (28%) [4]. The existing literature highlights numerous factors of vaccination intent, such as perceived risks, benefits, and costs of vaccination [18-22]; social and media communication on vaccines [21-25]; and advertising [26,27]. Tough measures to increase the uptake level based on coercion or tangible incentives are problematic. Specifically, imposing mandatory COVID-19 vaccinations faces considerable objections (eg, half of the German adults were against mandatory vaccinations in 2020) [28]. The effectiveness of promoting vaccinations through monetary incentives is shown to be limited [29]. Therefore, it is crucial to promote vaccinations by informing the public about them [1] through mass and personal communication, training, and education [30]. Various new media and technologies have been used to convey vaccine-related messages in a large-scale but personalized way. Vaccine promotion organizations use websites and social media to share information and raise public awareness [31,32].

Chatbots can be accessible to many users and enable conversations with them, supporting scaling up and personalizing health communication [33,34]. In the context of addressing user doubts, chatbots can determine which of the most common counterarguments in a given population are endorsed by a given user and provide relevant rebuttals [33]. It is proposed [35] that chatbots can combine the knowledge of conversational norms and relational strategies with storing conversation history for a given user to scale up the relational capacity. Although people treat chatbots differently from humans (eg, by using shorter messages and poorer vocabulary [36]), such conversations may proxy personal communication. This may ensure user engagement [12] and responsiveness to users' doubts [1,33]. Moreover, people tend to consider machines more objective and less biased than humans (“machine heuristic” [37]), which may be advantageous for chatbots versus humans in health communication. Chatbots may inform on and change COVID-19–preventive behaviors [38]. Altay et al [1] demonstrated that COVID-19 vaccination intention increased after interacting with a chatbot responding to counterarguments. Parents provided with vaccine information by chatbots were more in favor of children's vaccinations [39]. Amiri and Karahanna [2] reported over 20 publicly accessible chatbots that provide virus and vaccine education, some of which offer misinformation/disinformation fact checking. However, the quality of information that vaccination chatbots provide is considered a cause for concern [40].

#### The Pitfall of Expressing Empathy/Autonomy Support Using Chatbots

In the context of vaccination, promotion may be considered as limiting one's freedom. Namely, convincing or persuading someone to vaccinate leads the communication receiver to perform an activity (ie, vaccination) that may be perceived as intrusive and influential on their body and health. Thus, psychological reactance, a motivational state oriented toward reestablishing one's freedom when threatened [8], may play a key role in responding to vaccine communication. Displaying empathy in health communication, that is, showing the recognition of the receivers' experience, may make them feel supported [41]. Specifically, by taking the receiver’s perspective, health communication becomes less pushy and perceived as more internal, which may lead to a higher perceived autonomy of the receiver [42]. Therefore, we consider expressing empathy and supporting autonomy (denoted as “expressing empathy/autonomy support”) as an internally coherent property of a chatbot. This property can be manifested in a conversation by various expressions suggesting that a chatbot understands its users, takes their perspective, and stands by their side instead of pushing them. As posited by reactance theory [8], granting autonomy by enhancing the freedom of choice in communication may diminish the receivers' reactance. Health communication using less controlling language and taking the receivers' perspectives by highlighting that they are free to decide what is best for them was demonstrated to evoke less negative reactions, such as less anger and a lower perceived threat to freedom, respectively [7].

Chatbots may differ in expressing empathy/autonomy support, whose influence on the user response appears complex. The Computers as Social Actors (CASA) approach [43,44] posits that people tend to perceive their interactions with machines (eg, chatbots) as interactions with other people. As empathy may be considered an anthropomorphic dimension of conversational agents [12], one may expect that expressing empathy/autonomy support using chatbots produces positive user reactions. Accordingly, De Gennaro et al [9] showed that the effect of experienced social exclusion was less negative when participants interacted with an empathic chatbot instead of an interactive questionnaire. The chatbot’s empathy manifested itself in its responses to the participants mentioning their exclusion in social media. The chatbot expressed understanding, felt sorry, and comforted the participants. However, the Uncanny Valley of Mind (UVM) theory [11] posits that a machine that is too similar to humans may evoke negative reactions (eg, by perceiving it as eerie). Liu and Sundar [6] demonstrated both positive and negative effects of chatbot empathy on perceived chatbot eeriness. In their experiment, the participants read a predefined script of the conversation between a user and a chatbot. The experimental conditions differed in the form of empathy expressed by the chatbot, and the chatbot expressed no empathy in the advice-only control condition. Suggesting that the chatbot felt sorry for the users’ problems was negatively related to the perceived eeriness, but suggesting that the chatbot understood users’ feelings about their problems was positively related to the perceived eeriness. Similar negative effects of empathy have been demonstrated for other artificial agents. For example, Stein and Ohler [11] reported that users perceived an empathic virtual reality avatar as eerier. A robot expressing a higher (vs a lower) ability to infer the mental states of its users was demonstrated to be perceived as creepier [10]. More generally, in some studies, the anthropomorphic robot features were demonstrated to be negatively related to user attitudes [12,45], and emotions displayed by chatbots may harm user autonomy [46].

The existing evidence on the consequences of chatbot empathy provides no conclusion regarding user reactions to vaccine promotion chatbots, including CPI and chatbot persuasiveness (ie, the degree to which user vaccination intent changes after the conversation with the chatbot). Furthermore, although previous studies suggest that the way of empathizing (ie, too “humanlike” expression) may lead to a negative response, it is unclear how the response to vaccination chatbot empathy depends on the context related to the conversation, such as the conversation quality and chatbot expertise cues (eg, the chatbot’s endorser credibility). Conversation quality is the actual quality of the information provided by a chatbot to specific users' questions, which is considered a key component of chatbot performance [47]. Chatbot expertise cues may increase initial trust toward the chatbot [16] and, therefore, act as an important factor in the response to its empathy/autonomy support expression. This gap is also relevant for vaccine policy makers and developers of vaccination chatbots as both conversation quality and chatbot expertise cues are directly manageable. To bridge this gap, we aim to investigate how the relationship between the user response to COVID-19 vaccination chatbots and the chatbots’ expressed empathy/autonomy support depends on conversation quality and the chatbots’ endorser credibility.

We propose that the effect of vaccination chatbot empathy/autonomy support expression on user response may be moderated by the conversation quality. In general, the quality of chatbot performance consists of how well a chatbot matches its predefined responses with the user’s request and how well the set of the chatbot’s predefined responses covers the requests of its users [48]. Accordingly, chatbot performance at the conversation (or interaction) level may be indicated by the relevance of provided answers versus user requests, the presence of predefined chatbot responses and questions versus user requests, and the (low) frequency of failures (ie, instances when a chatbot cannot provide a response to a user request). Anthropomorphizing conversation agents and service agents may raise user expectations related to the quality of interaction [12,13] and thus make users more sensitive to interaction problems, as suggested by Pardo et al [49]. Here, one may refer to incongruity theory [50,51], positing that unresolved incongruity between stimuli (here the expressed empathy/autonomy support and the actual performance of the chatbot) may lead to negative reactions. In other words, users may react less positively (or even more negatively) to a chatbot's declarations of its empathy/autonomy support when they are incongruent with the actual quality of the output of the chatbot. Therefore, it seems plausible that the effect of vaccination chatbot empathy/autonomy support expression on the user response is less positive (or more negative) when the actual answers provided by the chatbot are of lower quality (eg, when the chatbot responds that it does not understand the user and it cannot answer their question). Considering this, our research aims to test the following:

Hypothesis (H)1: The relationship between the user response to COVID-19 vaccination chatbots (ie, CPI and chatbot persuasiveness in terms of COVID-19 vaccination) and their expressed empathy/autonomy support is less positive (or more negative) when the conversation quality is lower.

Chatbot endorser credibility may be an additional moderator of the chatbot empathy/autonomy support expression effect. Namely, a more credible endorser (ie, expert vs layperson) may act as an expertise cue and therefore produce a higher level of initial trust toward the chatbot [16]. Then, a more positive response to the chatbot empathy/autonomy support expression may be plausible as being more consistent with that initial attitude. Formally:

H2: The relationship between the user response to COVID-19 vaccination chatbots (ie, CPI and chatbot persuasiveness in terms of COVID-19 vaccination) and their expressed empathy/autonomy support is more positive (or less negative) when the chatbot is endorsed by an expert (vs a layperson).

Our experimental study aims to test user reactions to COVID-19 vaccination chatbots with different levels of expressed empathy/autonomy support, examining the proposed moderating role of conversation quality and chatbot endorser credibility.

## Methods

### Sample

In total, 211 Dutch-speaking adults living in Belgium (aged 18-60 years) and not vaccinated (yet) participated in an online experiment. The participants were panel respondents of the market research company Bilendi, Belgium. Their panel was recruited through various sources, such as the Bilendi website, banner media websites, member-get-member, third-party databases, and offline recruitment. To join the panel, respondents had to complete a sociodemographic questionnaire, approve a double opt-in where they confirmed they wanted to participate in Bilendi’s surveys, and accept their privacy policy. The participants were approached for the survey through personal email invitations and reminders. To thank the respondents for their contribution and encourage them to complete the online experiment, Bilendi granted points that the respondents could save up and swap for a prize later. The data collection was conducted in 2021, between May 28 and June 3, while daily new cases in Belgium were around 1400 [52], and around 54% of the adult population in Flanders and Brussels (where Dutch is an official language) were vaccinated against COVID-19 with at least 1 dose [53]. After the preintervention survey, the participants were redirected to the chatbot conversation (ie, the intervention) and then redirected to the postintervention survey. Due to logging problems, the responses of 15 (7.1%) participants could not be linked back to their IDs. Specifically, in the postintervention survey, all the participants were asked to manually fill out a code generated after their conversation with the chatbot. In 15 (7.1%) cases, a blank, false, or nonexisting code was filled out. Therefore, those responses could not be linked to the participants’ conversations. In similar future experiments, the logging should be automated to prevent such kind of data loss. Therefore, the final sample consisted of 196 (92.9%) participants (mean age 39.4, SD 9.4 years; n=112, 57.1%, females; see the sample characteristics in Table 1). At the time of surveying, 63.8% (n=125) of our participants had not yet received an invitation to be vaccinated against COVID-19, 33.7% (n=66) were invited and waited for the first shot, and 2.6% (n=5) were invited and refused. The vast majority of participants (n=186, 94.9%) declared they had not contracted COVID-19.

**Table 1:** Sample characteristics (N=196).

Characteristics		Total sample, n (%)	Present empathy/autonomy support expression, n (%)	Absent empathy/autonomy support expression, n (%)
**Gender**				
	Women	112 (57.1)	64 (63.4)	48 (50.5)
	Men	84 (42.9)	37 (36.6)	47 (49.5)
**Age (years)**				
	<26	14 (7.1)	8 (7.9)	6 (6.3)
	26-35	60 (30.6)	37 (36.6)	23 (24.2)
	36-45	64 (32.7)	32 (31.7)	32 (33.7)
	46-55	55 (28.1)	24 (23.8)	31 (32.6)
	>55	3 (1.5)	N/A^a^	3 (3.2)
**Education**				
	Primary	1 (0.5)	1 (1.0)	N/A
	Secondary	40 (20.4)	13 (12.9)	27 (28.4)
	Bachelor’s degree	81 (41.3)	44 (43.6)	37 (38.9)
	Master’s degree	71 (36.2)	41 (40.6)	30 (31.6)
	Doctoral degree	3 (1.5)	2 (2.0)	1 (1.1)

^a^N/A: not applicable.

### Stimuli

As experimental stimuli, we used VaccinChat, a chatbot providing COVID-19 vaccination information. VaccinChat was developed by the CLiPS (Computational Linguistics, Psycholinguistics and Sociolinguistics) research center associated with the Department of Linguistics of the Faculty of Arts of the University of Antwerp, Belgium. The chatbot is a RASA [54] artificial intelligence (AI)–equipped frequently asked questions (FAQ) chatbot, trained to classify user questions provided in open text fields into predefined answer intents, and it has been publicly available as a development version. It does not consider a specific conversation flow but answers questions in series. The final version is trained on 12,883 questions referring to 181 answers [55].

### Procedure

The experiment had a 2 (chatbot empathy/autonomy support expression: present vs absent) × 2 (endorser credibility: expert vs layperson) between-subject design. The participants were randomly assigned to the experimental conditions, however, striving for equal gender distribution. The final sample sizes were N_present_=101 and N_absent_=95 across the empathy/autonomy support expression condition and N_expert_=111 and N_layperson_=85 across the endorser credibility condition (experimental cell sizes: N_empathy/autonomy present, expert_=57, N_empathy/autonomy present, layperson_=44, N_empathy/autonomy absent, expert_=54, N_empathy/autonomy absent, layperson_=41). No significant differences in initial vaccination intention occurred across the experimental condition (empathy/autonomy support expression condition: *t*_194_=0.336, *P*=.74; endorser credibility condition: *t*_194_=0.719, *P*=.47). In addition, no significant differences occurred in demographics for empathy/autonomy support expression condition (gender: *χ*^2^_1_=3.296, *P*=.07; age range: *χ*^2^_4_=7.266, *P*=.12; education: *χ*^2^_4_=8.367, *P*=.08). For endorser credibility condition, there were no significant differences in age and education (age range: *χ*^2^_4_=1.628, *P*=.80; education: *χ*^2^_4_=1.719, *P*=.79), but the percentage of women was higher in the expert condition (n=71 vs 41, 64.0% vs 48.0%, *χ*^2^_1_=4.863, *P*=.03). To ensure the sample characteristics would not disturb the tested effects, we involved gender, age range, education, and initial vaccination intention as covariates in all analyses.

The participants first received an invitation mail with the survey link randomly redirecting them to the 4 experimental conditions. After the participant provided informed consent, the preintervention survey started with demographics and a question on COVID-19 vaccination status, initial COVID-19 vaccination intent, and personal experience with COVID-19. Next, the participants were informed about the chatbot, they received the related instructions and time recommendations, and they were redirected to the chatbot and asked to start the conversation. We encouraged the participants to submit their questions and concerns about COVID-19 vaccinations. The instruction was as follows:

In a moment, you will be asked to start a conversation with the chatbot. A chatbot is a kind of computer program. There is no real person behind the chat. The conversation will open in a new tab. We are interested in your experience with the chatbot. The idea is that you will submit any questions or concerns you may have about the COVID-19 vaccines and vaccination to the chatbot. These questions can be very practical but can also be about genuine concerns or serious vaccine doubts. If the chatbot does not understand your question, please try to word your question differently. We ask you to have a conversation with the chatbot for a minimum of 3 minutes and a maximum of 5 minutes.

Before starting a conversation with the chatbot, the participants read a brief endorsement claiming, “The chatbot gives a clear answer to the questions and concerns about the COVID-19 vaccines,” and that the chatbot is “particularly on point.” In the expert endorser condition, this endorsement was provided by “the well-known vaccinologist, Prof. Dr. Pierre Van Damme.” In the layperson endorsement condition, the endorsers were described as “a group of Flemings with questions about vaccines, including vaccine doubters.”

The chatbot worked differently depending on the empathy/autonomy support expression condition. Namely, in the present empathy/autonomy support expression condition, the chatbot displayed additional phrases to express its empathy and autonomy support. Specifically, the chatbot showed understanding of the user’s concerns (eg, “I understand your concern. This is ultimately about your own body.”), showed appreciation for the user’s knowledge (eg, “Good that you are aware of this.”), supported the user’s autonomy regarding the decision to get vaccinated (eg, “Whether or not you vaccinate your children, when possible, remains, of course, a personal choice.”), showed interest in the user’s situation (eg, “I hope with you that we can soon pick up life again in all its glory.”), and comforted the user by referring to the social context (eg, “There are many people who are currently facing this question.”). See all phrases appearing in the predefined set of the chatbot's answers in Table 2.

**Table 2:** Empathy/autonomy support expression phrases in the predefined set of the chatbot's answers (the present empathy/autonomy-support expression condition).

Way of expressing empathy/autonomy support	Phrases in the predefined set of the chatbot's answers (listed in alphabetical order)
Showing an understanding of the user’s concerns	“I understand your concern. This is ultimately about your own body.” “It is normal that you are curious about the differences. It is even good that you are aware of this.” “It is quite normal that you would prefer to select your vaccine yourself. Maybe you still have certain questions about some vaccines? Do not hesitate to ask them.” “We understand your concerns about possible side effects of a vaccine.”
Showing appreciation for the user	“Good question!” “Good that you are aware of this.” “Good that you are checking this out. I will try to make it clear to you below.” “Good that you are informing yourself about the different vaccines currently being used.” “Good that you are inquiring about this.” “How nice to read that you want to inform yourself well about the moment when it is your turn for a vaccine.” “So it's good that you think about this.”
Expressing autonomy support regarding the decision to get vaccinated	“Getting vaccinated, therefore, remains entirely your own choice. List for yourself which considerations are important to you. This can help you choose whether or not you want to vaccinate.” “Whether or not you vaccinate your children, when possible, remains, of course, a personal choice.” “I am happy to help you find reliable information about vaccines. The final choice to get vaccinated is entirely up to you.”
Showing interest in the user’s situation	“I hope you can get your vaccine soon, if desired!” “I hope with you that we can soon pick up life again in all its glory.”
Comforting the user by referring to the social context	“There are many people who are currently facing this question.”

These phrases (Table 2) were removed in the absent empathy/autonomy support expression condition.

The empathy/autonomy support phrases were attached to predefined chatbot answers (called adapted answers). The occurrence of those answers in a conversation depended on the questions asked by each participant. In the actual conversations made with the participants in our study, the mean share of the adapted answers (ie, the ones belonging to the predefined set of the chatbot's answers differing between the high and low empathy/autonomy support expression condition) was 63% (SD 0.2349). To ensure that the share of adapted answers for empathy/autonomy support expression at the participant level would not disturb the tested effects, we involved it as an additional covariate in all analyses.

The most frequently asked questions were similar to those reported by Buhmann et al [55], such as out-of-domain questions, general COVID-19 questions that do not consider vaccination (eg, regulations), questions about side effects of the vaccines, and questions about who will get their vaccine when and where. User questions that deal with trust issues mainly focused on vaccines in general, science, and governmental institutes, and policies. The average number of user questions was similar across the empathy/autonomy support condition (mean_present_ 8.181, SD 7.364; mean_absent_ 8.787, SD 9.598; *F*_1,187_=0.232, *P*=.63) and the endorser credibility condition (mean_expert_ 8.022, SD 9.688; mean_layperson_ 8.945, SD 6.674; *F*_1,187_=0.548, *P*=.46). In addition, the average length of the user questions was similar across the conditions (empathy/autonomy support condition: mean_present_ 8.327, SD 4.403; mean_absent_ 7.669, SD 3.043; *F*_1,187_=1.401, *P*=.24; endorser credibility condition: mean_expert_ 7.633, SD 3.021; mean_layperson_ 8.363, SD 4.637; *F*_1,187_=1.752, *P*=.19).

After their conversation with the chatbot, the participants had to use the conversation code they received from the chatbot at the beginning of the conversation to start the postintervention survey. Based on that code, we were able to link their answers to their IDs. Next, we measured the participants’ perceived autonomy during the conversation, CPI, and the intention to get vaccinated against COVID-19. They were debriefed that the chatbot gave different responses to different participants, but the information provided by the chatbot did not contain any factual falsehoods. Finally, the participants were asked for feedback, thanked, and received monetary compensation.

### Measurements

#### Vaccination Intention Shift

The initial COVID-19 vaccination intention was measured right after the question about the COVID-19 vaccination status with the item “If you had to make a choice now, will you get vaccinated or not, as soon as the opportunity presents itself?” The after-conversation intention was measured with the item “After talking to the chatbot, will you get vaccinated against COVID-19 as soon as you get an invitation?” Both items used a 6-point response format ranging from 1 (very unlikely/certainly not) to 6 (very likely/certainly so). The vaccination intention shift (VIS) was determined by subtracting the initial COVID-19 intention from the after-conversation intention and coded from –5 (indicating the highest-possible decrease) to 5 (indicating the highest-possible increase).

#### Perceived User Autonomy

Here, 10 items adapted from Smit et al [56] measured the perceived support for user autonomy (perceived user autonomy [PUA]) in a virtual care setting (here, during the conversation with the chatbot; α=.905). The statements were related to giving freedom of choice (eg, “I feel that the conversation with the chatbot has given me choices and options.”) and empathy (eg, “I felt understood during the conversation with the chatbot.”); see details in Table 3. The items used a 5-point response format ranging from 1 (do not agree at all) to 5 (strongly agree).

**Table 3:** Measurement scales.

Scale	Item	Reliability, α	Reference
PUA^a^	“I feel that the conversation with the chatbot has given me choices and options.” “I felt understood during the conversation with the chatbot.” “I felt that the chatbot was not judging me during the conversation.” “The conversation with the chatbot encouraged me to look for answers to my questions.” “I have great confidence in the chatbot.” “The chatbot answered my questions completely and carefully.” “During the conversation with the chatbot, I was able to give input on how I would like to do things myself.” “The chatbot took into account my emotions in the answers and advice given.” “I felt that the chatbot cares about me as a person.” “The chatbot tried to include how I see things in the advice given.”	.905	Adapted from Smit et al [56]
CPI^b^	“I am willing to submit questions I have about vaccines to the chatbot.” “The likelihood that I would ask the questions I have about vaccines to the chatbot is high.” “I am willing to recommend the chatbot to friends.”	.921	Similar to Zarouali et al [57]

^a^PUA: perceived user autonomy.

^b^CPI: chatbot patronage intention.

#### Chatbot Patronage Intention

Here, 3 items measured the patronage intention toward the chatbot (CPI; α=.921). Similar items have been frequently used in previous research (eg, [57]). The statements were related to the willingness and likelihood of reusing and recommending the chatbot (see details in Table 3). The items used the same response format as in the PUA measurement.

#### Chatbot Conversation Quality

In addition to the respondents' perceptions, we analyzed the quality of actual answers provided by the chatbot for each respondent using the following metrics based on conversation logs:

- Conversation accuracy (CA): The percentage of correct answers in the conversation (an answer's correctness was determined manually by assessing the relevance of the answer provided by the chatbot to the user's question). The mean CA was 53.0% (SD 0.24295); 100% CA was reached for 9.2% (n=18%) of the participants, and it exceeded 50% for 45.4% (n=89) of the participants.
- Conversation-in-database (CD): The percentage of answers in the conversation that were exactly the same as questions that were already included in the chatbot training data. The mean CD was 5.9% (SD 0.11294); for 70.4% (n=138) of the participants, the CD was 0% (no exact answers), and it did not exceed 50% for the other participants.
- CF: The percentage of answers in the conversation that took the form of ”I don't understand,“ followed by a request to rephrase the question. The mean CF was 4.0% (SD 0.08979); the CF was 0% for 77.6% (n=152) of the participants (no fallbacks), and it did not exceed 50% for 99.5% (n=195) of the participants.
- Conversation wrong-answer (WN): The percentage of user questions in the conversation for which the chatbot did not have a suitable answer and provided an answer that corresponded to another question. The mean WN was 12.5% (SD 0.17254); the WN was 0% for 52.6% (n=103) of the participants (no wrong answers), and it did not exceed 50% for 97.4% (n=191) of the participants.

Most errors could be attributed to questions that were not yet included in the chatbot at the moment of the intervention. For example, a number of participants asked questions about the possibility of a third vaccination round because this had been reported in the media. The chatbot, however, had not been updated to deal with this information at that time.

### Ethical Considerations

The Ethics Committee for the Social Sciences and Humanities of the University of Antwerp, Belgium (code of ethical approval: SHW_21_57) approved this research. Prior to the study, all respondents signed the informed consent form. All data were anonymously collected; we only kept track of an assigned response ID. All human experimentation guidelines were respected. Our participants received monetary compensation for their contribution to our online experiment.

## Results

### Effects of Different Conditions

First, we ran 2 ANCOVAs to check the main effects of chatbot empathy/autonomy support expression on chatbot evaluation separately for PUA and CPI as dependent variables. The empathy/autonomy support expression condition (present vs absent) and the endorser credibility condition (expert vs layperson) were factors, and the share of adapted answers for empathy/autonomy support expression, gender, age, education, and initial vaccination intention were covariates. No significant manipulation effects occurred under the following conditions:

- PUA under the empathy/autonomy support expression condition: mean_present_ 2.935, SD 0.747; mean_absent_ 3.045, SD 0.864; *F*_1,187_=0.906, *P*=.34
- PUA under the endorser credibility condition: mean_expert_ 2.983, SD 0.858; mean_layperson_ 2.996, SD 0.736; *F*_1,187_=0.012, *P*=.91
- CPI under the empathy/autonomy support condition: mean_present_ 3.150, SD 1.152; mean_absent_ 3.450, SD 1.289; *F*_1,187_=3.023, *P*=.08
- CPI under the endorser credibility condition: mean_expert_ 3.293, SD 1.233; mean_layperson_ 3.306, SD 1.223; *F*_1,187_=0.006, *P*=.94

In addition, the 2 manipulations had no interaction effects (for PUA: *F*_1,187_=0.735, *P*=.39; for CPI: *F*_1,187_=0.327, *P*=.57), providing no support for H2.

**Figure 1:**
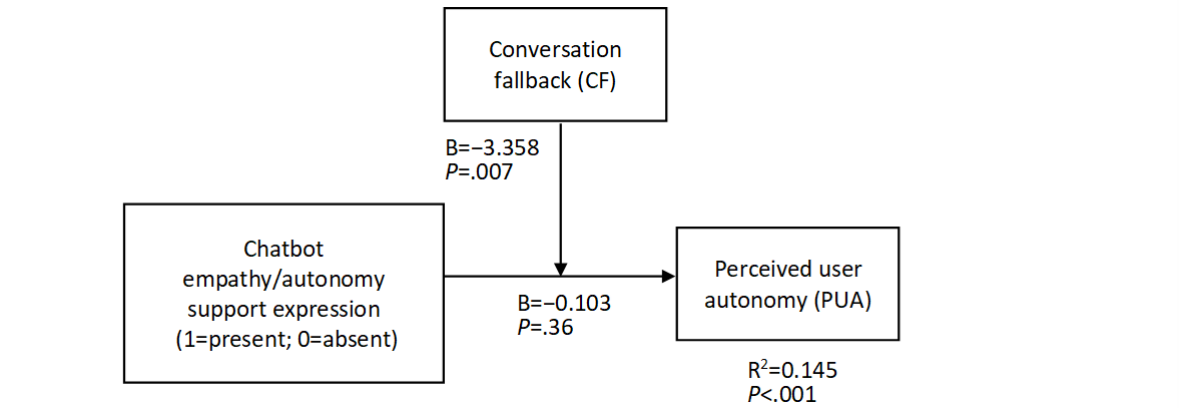
Moderation effects of CF in the relationship between chatbot empathy/autonomy support expression and PUA.

**Figure 2:**
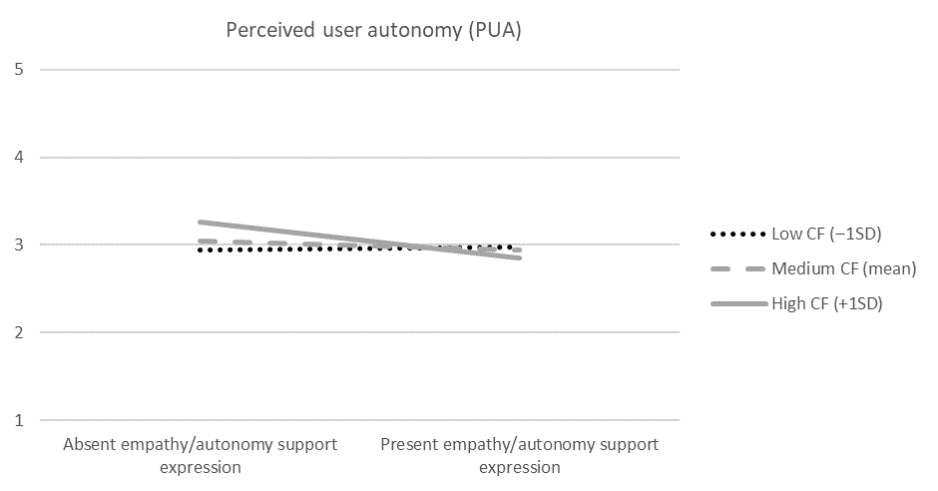
Visualization of conditional effects of chatbot empathy/autonomy support expression on PUA (CF is the moderator). CF: conversation fallback.

Next, we checked the role of actual answers provided by the chatbot in the relationship between the empathy/autonomy support expression and chatbot evaluation. Specifically, we checked the moderation effects of conversational quality variables. We ran a series of moderation analyses (PROCESS macro [58], model 1) with the empathy/autonomy support expression condition (dummy-coded as 1=present, 0=absent) as an independent variable, PUA as a dependent variable, and a conversation quality variable as a moderator. The endorser credibility condition (dummy-coded as 1=expert, 0=layperson), the share of adapted answers for empathy/autonomy support expression, gender (dummy-coded as 1=women, 0=men), age, education, and initial vaccination intent served as covariates. We included the endorser credibility condition as a covariate to ensure the differences in its share between the empathy/autonomy support expression condition would not disturb the tested effects. No moderation effects occurred with the following moderators: CA (B=0.396, SE 0.434, *t*_186_=0.911, *P*=.36), CD (B=–0.119, SE 1.154, *t*_186_=0.103, *P*=.92), and WN (B=–0.362, SE 0.679, *t*_186_=.533, *P*=.59). However, in the moderation model with CF as a moderator (Figure 1; *R*^2^=0.145, *F*_9,186_=3.507, *P*<.001, variance inflation factors [VIFs]<1.2), the interaction effect of CF and empathy/autonomy support expression was negative (B=–3.358, SE 1.235, *t*_186_=2.718, *P*=.007), in line with H1. The conditional effect of empathy/autonomy support expression at the CF level of +1SD was negative (Figure 2; B=–0.405, SE 0.158, *t*_186_=2.564, *P*=.011), while being nonsignificant at the mean level (B=–0.103, SE 0.113, *t*_186_=0.914, *P*=.36) and the –1SD level (B=0.031, SE 0.123, *t*_186_=.252, *P*=.80). The Johnson-Neyman cutoff value of CF was 4.0% (Table 4). The conditional effects of empathy/autonomy support expression at CF levels below the cutoff were nonsignificant (*P*>.10), while for levels above the cutoff, the effects were significantly negative (*P*<.05). These results indicate that when CF is higher than 4.0%, empathy/autonomy support expression decrease PUA, while empathy/autonomy support expression does not have an impact on PUA when CF is lower than 4.0%.

We used CF as a moderator in further analysis. Aiming to check the consequences of the CF × empathy/autonomy support expression interaction on behavioral outcomes, we ran 2 first-stage moderated mediation analyses (PROCESS macro [58], model 7, 5000 bootstrap samples). Both analyses used the empathy/autonomy support expression condition (dummy-coded as 1=present, 0=absent) as an independent variable, PUA as a mediator, and CF as a first-stage moderator. The set of covariates was the same as in the former analysis (VIF<1.2).

**Figure 3:**
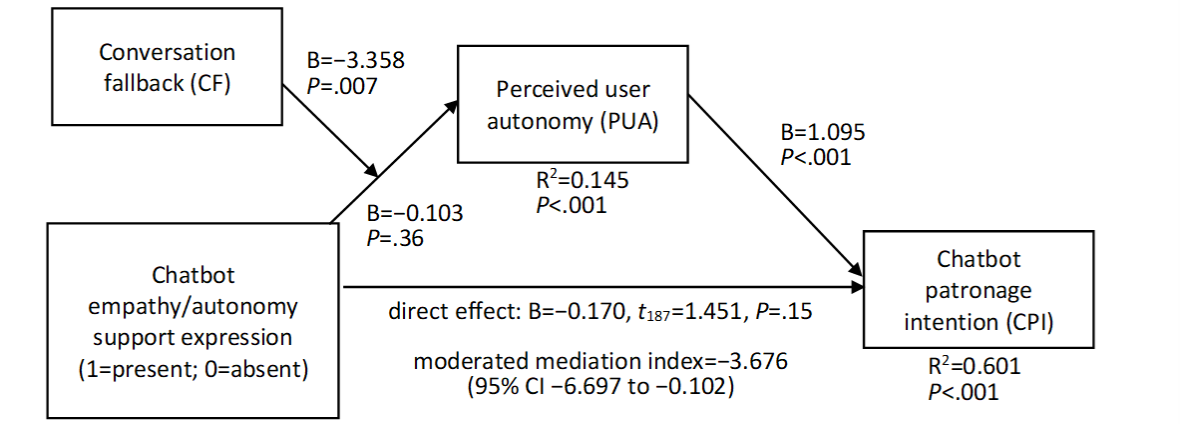
Moderated mediation effects on CPI. CPI: chat patronage intention.

In the first analysis, CPI served as a dependent variable (Figure 3; *R*^2^=0.601, *F*_9,187_=35.237, *P*<.001). The effect of PUA on CPI was positive (B=1.095, SE 0.074, *t*_187_=14.719, *P*<.001), and the moderated mediation index was negative (index=–3.676, BootSE 1.614, 95% CI –6.697 to –0.102). The conditional indirect effect of empathy/autonomy support expression on CPI at the CF level of +1SD was negative (B=–0.443, BootSE 0.202, 95% CI –0.809 to –0.005), while 95% CIs included 0 at the mean level (B=–0.113, BootSE 0.124, 95% CI –0.346 to 0.137) and the –1SD level (B=0.034, BootSE 0.132, 95% CI –0.224 to 0.305).

**Figure 4:**
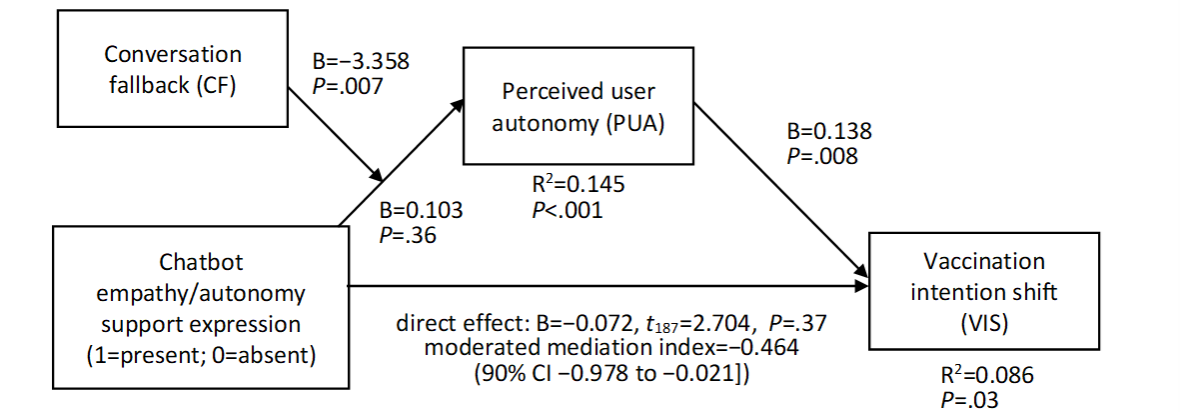
Moderated mediation effects on VIS. VIS: vaccination intention shift.

In the second analysis, VIS served as a dependent variable (Figure 4; *R*^2^=0.086, *F*_9,187_=2.194, *P*=.03). The effect of PUA on VIS was positive (B=0.138, SE 0.051, *t*_187_=2.704, *P*=.008), and the moderated mediation index was marginally negative (index=–0.464, BootSE 0.302, 90% CI –0.978 to –0.021). The conditional indirect effect of empathy/autonomy support expression on VIS at the CF level of +1SD was marginally negative (B=–0.056, BootSE 0.036, 90% CI –0.118 to –0.002), while being nonsignificant at the mean level (B=–0.014, BootSE 0.018, 90% CI –0.044 to 0.014) and the –1SD level (B=0.004, BootSE 0.019, 90% CI –0.023 to 0.039).

**Table 4:** Johnson-Neyman output for the moderation effect of CF^a^ in the relationship between chatbot empathy/autonomy support expression (independent variable; 1=present, 0=absent) and PUA^b^ (dependent variable).

CF	Effect	SE	*t*_188_	*P* value	95% CI
–0.0399	0.0310	0.1232	0.2517	.80	–0.2121 to 0.2742
–0.0121	–0.0623	0.1138	–0.5472	.59	–0.2867 to 0.1622
0.0157	–0.1555	0.1143	–1.3613	.18	–0.3810 to 0.0699
0.0421	–0.2445	0.1239	–1.9728	.05	–0.4889 to 0
0.0434	–0.2488	0.1246	–1.9972	.047	–0.4946 to –0.0030
0.0712	–0.3421	0.1426	–2.3985	.02	–0.6235 to –0.0607
0.099	–0.4354	0.1659	–2.6245	.009	–0.7627 to –0.1081
0.1268	–0.5287	0.1925	–2.7465	.007	–0.9085 to –0.1489
0.1545	–0.622	0.2212	–2.8114	.006	–1.0584 to –0.1855
0.1823	–0.7153	0.2514	–2.8455	.005	–1.2112 to –0.2194
0.2101	–0.8086	0.2825	–2.8625	.005	–1.3658 to –0.2513
0.2379	–0.9018	0.3142	–2.8700	.005	–1.5217 to –0.2819
0.2657	–0.9951	0.3465	–2.8721	.005	–1.6787 to –0.3116
0.2934	–1.0884	0.3791	–2.8711	.005	–1.8363 to –0.3405
0.3212	–1.1817	0.4120	–2.8683	.005	–1.9945 to –0.3689
0.349	–1.2750	0.4451	–2.8645	.005	–2.1531 to –0.3969
0.3768	–1.3683	0.4784	–2.8603	.005	–2.3120 to –0.4245
0.4045	–1.4616	0.5118	–2.8558	.005	–2.4712 to –0.4519
0.4323	–1.5548	0.5453	–2.8513	.005	–2.6306 to –0.4791
0.4601	–1.6481	0.5789	–2.8469	.005	–2.7902 to –0.5060
0.4879	–1.7414	0.6126	–2.8425	.005	–2.9500 to –0.5328
0.5157	–1.8347	0.6464	–2.8384	.005	–3.1099 to –0.5595

^a^CF: conversation fallback.

^b^PUA: perceived user autonomy.

## Discussion

### Principal Findings

The aim of our study was to improve the understanding of the role of the conversation-related context in the effects of expressing empathy/autonomy support using COVID-19 vaccination chatbots. Specifically, we checked the moderating role of 2 conversation context factors (ie, conversation quality and chatbot expertise cues). The results of our experiment suggest that chatbot empathy/autonomy support expression may harm user evaluation and behavioral intentions related to a chatbot and diminish its usefulness in promoting COVID-19 vaccinations when the conversation quality is low (as indicated by the higher levels of CF). However, we did not find the effects of chatbot expertise cues manipulated through chatbot endorser credibility.

We demonstrated that the effect of expressing empathy/autonomy support using the COVID-19 vaccination chatbot on PUA was negative for a high level of CF, while being nonsignificant for its mean and low levels. Moreover, we showed that this moderation effect has consequences on CPI and chatbot persuasiveness. The indirect effects of empathy/autonomy support expression on CPI and VIS through PUA were negative for a high level of CF, while being nonsignificant for its mean and low levels. These results add to the growing literature on the user response to chatbots (eg, [6,9,11,33,45,46,49]). Our results are in line with numerous previous studies indicating mixed or negative effects of chatbots' empathy [6,11], robots' capability to infer human mental states [10], robots' and conversational agents' anthropomorphic features [12,45], and chatbots' displayed emotions [46] on users' response to chatbots. Importantly, unlike these previous studies, our study was based on the measurement of the actual quality of conversation with a chatbot user instead of presenting cues or scenarios to study participants.

CF (ie, the chatbot replying ”I do not understand“) may represent the deficiency of actual chatbot empathy and, in turn, its limited support for user autonomy as the chatbot fails to resolve the request of the user. As such, higher levels of CF may indicate to the chatbot users that the chatbot's actual answers lack empathy and support for user autonomy. Considering this, the negative interaction of chatbot empathy/autonomy support expression and CF that we revealed is in line with the notion that chatbot anthropomorphism (here, in the form of empathy/autonomy support expression) may increase user expectations and sensitiveness for the quality of interaction [12,49]. Namely, a higher CF indicates an issue with empathy and support for user autonomy in actual chatbot answers, and users are more sensitive to this issue when chatbot empathy/autonomy support expression is higher. Although Pelau et al [59] demonstrated the positive relationship between perceived AI empathy and perceived AI interaction quality, we discerned the negative interaction effects of manipulated chatbot empathy/autonomy support expression and the actual conversation quality. Put differently, when CF is higher, the higher empathy/autonomy support expression increases the incongruity in empathy/autonomy support between the chatbot's actual answers and its declarations (expressions). This incongruity may produce negative user reactions [50,51]. This way, we shed new light on the existing chatbot research focusing on incongruities in chatbot perception [60]. Particularly, our results highlight that the positive effects of chatbot empathy, suggested by the CASA approach [43,44], diminish not only when a chatbot is too humanlike, as proposed by UVM theory [11], but also in the presence of incongruity between the declared empathy and the chatbot’s actual outputs.

In terms of Sundar's [61] framework of human-AI interactions, the incongruity in empathy/autonomy support between the actual answers and the chatbot’s declarations may represent the discrepancy between the cue route (based on visible attributes of the AI system, such as empathy expression) and the action route (based on the effects of human-AI collaboration, such as the actual quality of conversation). This way, our results suggest that those 2 routes may influence the user response to an AI-based system not only separately but also via their interplay. We applied these chatbot communication considerations to vaccination intention, showing that this incongruity may harm the persuasiveness of vaccination chatbots. This way, we have contributed to the literature on vaccination promotion through chatbots (eg, [1,2,39]).

No interaction effects of expressing empathy/autonomy support and the chatbot’s endorser credibility might be caused by our experimental setting, which involved extensive real interactions with the chatbot. In this case, the user response might be more influenced by the actual quality conversation, and therefore, expertise cues were discounted. Interestingly, endorser credibility also had no significant main effect on the user response.

### Limitations

Certain limitations of our study call for further research. First, our study used the Dutch-speaking population of Belgian adults who are demonstrated to have relatively positive attitudes toward vaccinations (eg, 84.0% agreement with ”Vaccines are effective“ [62]). In our sample, the majority of the respondents (77.8%) declared to be ”very likely“ to get vaccinated against COVID-19 as the initial intention. This might produce a ceiling effect and thus limit the observed effects of our chatbot on VIS. Therefore, future studies should cover populations with more negative vaccine attitudes (eg, Latvian adults, with 70.9% agreement with ”Vaccines are effective“ [62]).

Second, we were limited to a specific stage of the COVID-19 pandemic (ie, about 1 year after introducing the vaccines) and a specific preventable illness. Further research can extend the investigation of chatbot empathy/autonomy support effectiveness in later pandemic stages (eg, when COVID-19 became endemic) and other preventable illnesses (eg, influenza).

Third, we conceptualized and operationalized chatbot empathy and autonomy support together. Therefore, future studies examining the effects of chatbot empathy/autonomy -support expression should distinguish between those 2 constructs in experimental manipulation and measurements. Next, further studies may investigate deeper the mechanisms underlying the negative effects of the interaction of empathy/autonomy support expression and conversation quality, which were demonstrated in this paper. Specifically, whether those effects result from chatbot empathy being more likely to evoke an eerieness perception (as considered in UVM theory [11]) when the quality of the information provided by the chatbot is lower may be checked.

Fourth, we measured conversation quality instead of manipulating it. Although this approach enhances realism, as the quality of answers provided by chatbots may heavily depend on the interactions with their users, manipulating this variable should provide better causal evidence of its moderating role. Therefore, future experiments can compare conditions differing in the percentage of questions a chatbot fails to answer.

Lastly, our null result regarding the moderating role of expertise cues in the effect of expressing empathy autonomy support may result from our experimental setting, in which the participants were involved in extensive interaction with the chatbot. Perhaps, in the case of less engaging interactions, expertise cues play a larger role. This possibility should be examined in further research.

### Conclusion

Our study underlined the pitfalls of making vaccination chatbots empathetic in specific conversation-related contexts, namely too low conversation quality. The results may guide vaccine policy makers and chatbot developers to be careful with communicating chatbot empathy/autonomy support unless its capability to address user questions is firmly proven. Therefore, introducing a chatbot's empathy/autonomy support expression should be preceded by assessing the quality of answers provided by the chatbot. For example, in their early stage of development, chatbots with a high level of CF should hesitate to express their empathy and support for user autonomy. However, it is difficult to keep up with information flow manually when information changes quickly, like in a pandemic. Consequently, there may always be user questions for which the chatbot does not yet know appropriate answers, ending up with fallback. Automatic text-mining tools or a model, such as ChatGPT, could be used to scrape new information from the web more easily. In addition, an instruction model, such as ChatGPT, could provide a multitude of exemplary user questions. These could be used as extra training data, speeding up and improving the chatbot’s continuous growth, thus avoiding or reducing the number of fallback answers and, eventually, allowing the effective use of chatbot empathy/autonomy support expression.

## Abbreviations

AI: artificial intelligence
CA: conversation accuracy
CASA: Computers as Social Actors
CD: conversation-in-database
CF: conversation fallback
CPI: chatbot patronage intention
PUA: perceived user autonomy
UVM: Uncanny Valley of Mind
VIF: variance inflation factor
VIS: vaccination intention shift
WN: conversation wrong-answer

## Data Availability

The data set generated and analyzed during this study is available in the Open Science Framework, the repository of the Center for Open Science [63].
